# Quasi-Guided Modes in Titanium Dioxide Arrays Fabricated via Soft Nanoimprint Lithography

**DOI:** 10.1021/acsami.1c11456

**Published:** 2021-09-30

**Authors:** Jorge A. Garcia, Calin Hrelescu, Xia Zhang, David Grosso, Marco Abbarchi, A. Louise Bradley

**Affiliations:** † School of Physics and CRANN, Trinity College Dublin, Dublin 2, Ireland; ‡ CNRS, Aix-Marseille Université, Centrale Marseille, IM2NP, UMR 7334, Marseille 13013, France

**Keywords:** titanium dioxide, dielectric nanoresonator, nanoarrays, quasi-guided modes, metasurfaces, nanoimprint lithography

## Abstract

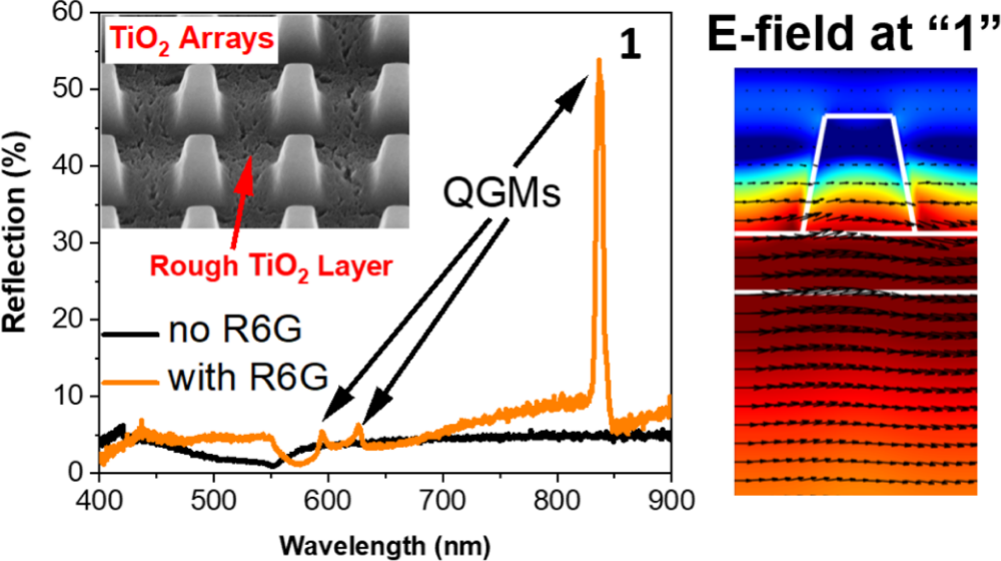

Reversible quasi-guided modes (QGMs) are observed in titanium dioxide (TiO_2_) metasurface arrays fabricated via soft nanoimprint lithography. A TiO_2_ layer between the nanopillar array and the substrate can facilitate the propagation of QGMs. This layer is porous, allowing for the tuning of the layer properties by incorporating another material. The presence of the QGMs is strongly dependent on the refractive index of the TiO_2_ layer. QGMs are not supported if the refractive index of the porous TiO_2_ is too low. It is demonstrated that after depositing R6G on the array QGMs can be observed as very strong and narrow reflectance peaks and transmittance dips. Furthermore, as the second material can penetrate through the pores into the layer it can experience the regions of high field enhancement associated with the QGMs. These results are of interest for a wide range of applications including but not limited to sensing, nonlinear optics, and emission control.

## 1 Introduction

Over the past two decades, there have been extensive studies in the field of metasurfaces using metallic and, more recently, dielectric nanoresonantors.^1^ Plasmonic metal nanoparticles suffer from high ohmic losses in the visible spectral range, which limits the quality factor (Q-factor) of the plasmonic resonances.^2,3^ Dielectric nanostructures, so-called “Mie resonators” or “dielectric resonators,” are an exciting alternative to plasmonic nanoparticles because of their lower ohmic losses in the visible range.^4−6^ Unlike metal nanoparticles, in which localized surface plasmons govern light–matter interactions, dielectric resonators trap light inside them, forming electric and magnetic resonant modes. These modes eventually leak out leading to significant scattering. Mie resonators can support electric and magnetic dipolar, quadrupolar, and higher order modes.^7^ The frequencies of these resonant modes depend on the size, shape, and refractive index, similar to the plasmon resonances of metallic nanoparticles.

The high ohmic losses associated with metal nanoparticles in the visible range inspired the search for low-loss plasmonic resonances in plasmonic metasurfaces. With ordered arrays of metallic nanoparticles, it is possible to have resonances with very high Q-factors when the period is roughly the wavelength of the incident light.^8^ This is achieved by the collective resonances of plasmonic arrays, for which the localized surface plasmons of the nanoparticles couple to the Rayleigh anomalies (RAs). These resonances are referred to as surface lattice resonances (SLRs).^8^ Because of the high Q-factors and sensitivity to the environment of SLRs,^9^ they have been considered for applications in enhancing the emission of dyes,^10,11^ light-emitting diodes,^12^ nonlinear optics,^13,14^ lasing,^15,16^ biosensing,^17^ and photovoltaics.^18^

With more recent focus on dielectric nanostructures, there has been growing interest in SLRs in dielectric arrays in the visible light range. In 2010, Evlyukhin et al. reported on the optical properties of 2D silicon arrays using the coupled dipole equations.^19^ SLRs in dielectric nanoarrays can be used for high-quality mirrors,^20^ the Kerker effect,^21^ electromagnetic-induced transparency,^22^ enhanced light emssion,^23,24^ and color generation surfaces.^25,26^ Very similar to SLRs, and often undistinguished in the literature,^27^ the individual pillar resonances in the arrays can couple into guided modes if the array is encased by a waveguide^23,27^ or on top of a waveguide.^22^ These modes are called quasi-guided modes (QGMs) as they are eventually scattered out by the particles.^14,28^ QGMs require a dielectric waveguide structure with a higher refractive index than the surrounding medium. Because of their high Q-factors, QGMs are also of interest for all the applications mentioned above.

Silicon has been widely used as a dielectric material for nanoresonators and metasurfaces in the visible and near-IR wavelength range. There have been reports showing the potential of Si metasurfaces for surface-enhanced Raman scattering,^29^ nonlinear effects,^30^ Kerker-type scattering,^4,31^ transverse scattering,^32^ and invisibility^33^ to name a few. However, the performance of silicon as a dielectric resonator reduces in the visible and near-UV region because of the increase in ohmic losses.^5,34^ A particularly interesting material is titanium dioxide (TiO_2_), as it exhibits near to zero losses in the visible range and it has a relatively high refractive index.^5,35,36^ TiO_2_ is a widely used material in many applications, from photocatalysis^37−40^ to cosmetics.^41^ Furthermore, TiO_2_ is used in the photodegradation of organic dyes as it increases the degradation rate.^42−44^ Apart from its optical properties, TiO_2_ is a material that is highly stable, light-weight, abundant, and safe for humans,^45^ making it a very appealing material for applications. TiO_2_ is an emerging material of interest for dielectric nanophotonics.^26,46−51^ Recently, fabrication techniques of TiO_2_ nanostructures have improved using soft nanoimprint lithography, which led to the fabrication of very large-area (>1 mm^2^) arrays with very good fidelity of particles and periodicity in the nanometer range.^46,47^ This is a cost-effective and scalable method for fabricating TiO_2_ metasurfaces. Furthermore, this technique is compatible with many different substrates and devices as the maximum temperature does not have to exceed 350 °C during fabrication. This temperature is enough to crystallize and stabilize the TiO_2_ into an anatase structure.^46,47^ For all these reasons, TiO_2_ is a very promising material for dielectric resonators. Although Mie resonances in TiO_2_ nanostructures have already been reported in the visible range in several studies,^26,46−51^ the potential of TiO_2_-based metasurfaces has not been fully explored yet.

This work reports on the experimental and numerical investigation of QGMs in TiO_2_ metasurface structures on top of a thin TiO_2_ layer on a glass substrate. Such structures can be fabricated using a variety of methods. Herein, we investigate structures fabricated via soft nanoimprint lithography according to the method described in refs (46, 47). Soft nanoimprint lithography is advantageous for fast large-area production of TiO_2_ metasurfaces, and the fact that it creates a thin TiO_2_ layer between the arrays and the substrate offers the potential to tailor the structure for QGMs without any other fabrication steps. We highlight the conditions under which QGMs can exist in TiO_2_ arrays. The nanopillar Mie resonances are in the visible range. Near-field simulations show the coupling of the electric and magnetic resonance of the nanopillars to the QGMs and the strong field enhancement in the TiO_2_ layer. It is shown how the porosity of the residual TiO_2_ layer effects the QGMs and that because of the porosity its refractive index can be tuned by introducing a new material into the pores. In this way, the presence of the QGMs can be controlled and is shown to be reversible. As the new material is incorporated into the layer, it can directly access the region of high field enhancement which is of interest for a wide range of applications exploiting enhanced light–matter interaction.

**Figure 1:**
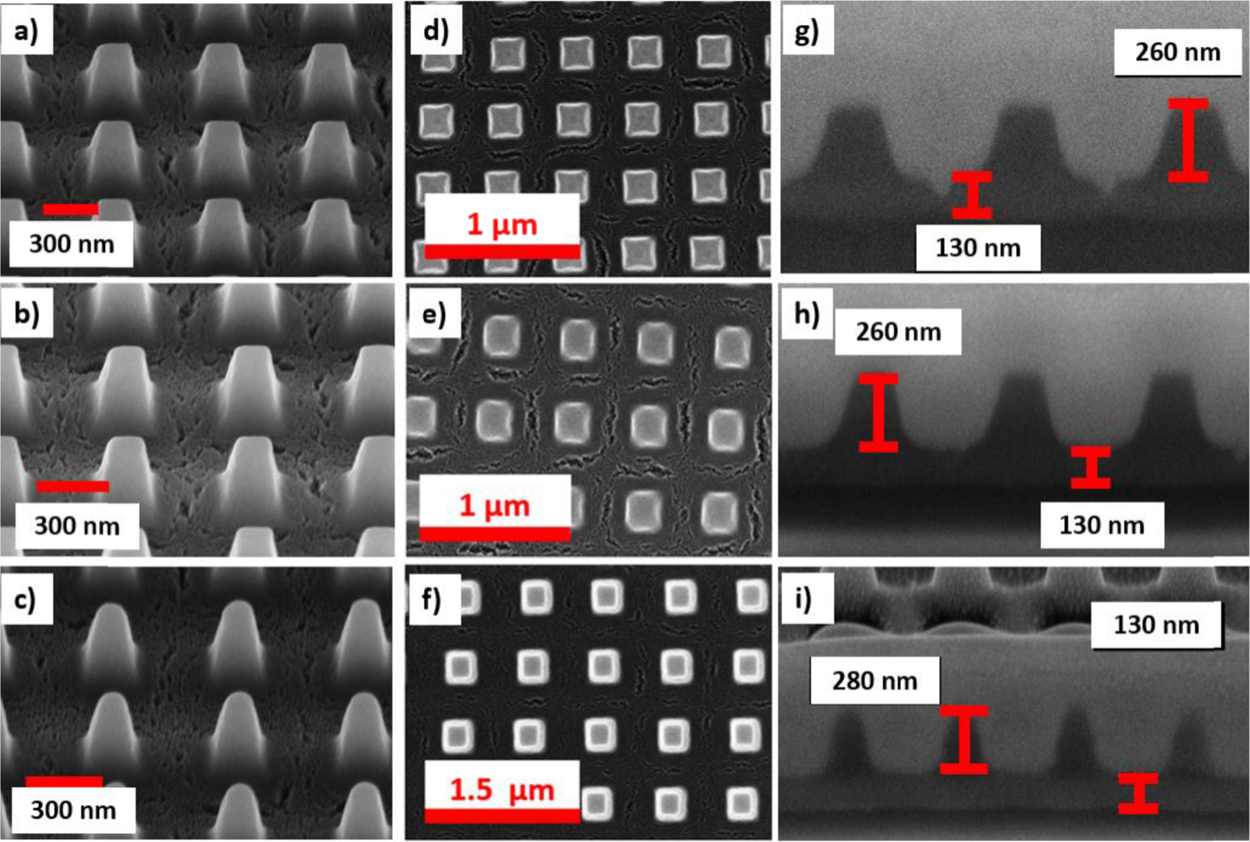
(a–c) Tilted SEM images of the A600, A550, and A480 arrays, respectively. (d–f) SEM images of the A600, A550, and A480 arrays, respectively. (g–i) SEM images of array cross-sections of the A600, A550, and A480, respectively. The cuts were performed by a focused-ion beam. The arrays on images a–c and g–i were tilted by 54°.

Three TiO_2_ arrays are experimentally investigated in this work, labeled A600, A550, and A480, respectively. They are fabricated together on the same glass substrate but differ from each other by periodicity and pillar dimensions. The square 2D arrays have an area of 1 mm^2^. Scanning electron microscopy (SEM) images of the arrays are shown in Figure 1. The dimensions of the arrays, measured using SEM imaging of focused-ion beam cuts of the sample, are given in Table 1. In Figure 1g–i, the thickness of the underlying TiO_2_ layer is approximately 130 nm. As can be seen, the TiO_2_ of this layer is more porous and rougher than that of the pillars. These latter features can be ascribed to relaxation of strain via formation of cracks after nanoimprinting, during the calcination step when the metal-oxide is annealed to remove the solvents.

**Table 1:** Dimensions of the Three Arrays^a^

array	period (nm)	base length (nm)	top length (nm)	residual layer thickness (nm)	height (nm)
A600	600	320	170	∼130	260
A550	550	280	150	∼130	260
A480	480	185	75	∼130	280

a The uncertainty in these measurements is ±10%.

The wavelengths corresponding to the RAs for rectangular arrays at normal incidence are calculated using the following equation:^53^
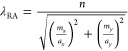
1where *a_x_* and *a_y_* are the lattice constants in the *x* and *y* directions. *m_x_* and *m_y_* are positive integers, and *n* is the refractive index of the environment. Because of the periodicity of these arrays, the RAs are in the visible spectral range. The first-order RAs from the air (a) and substrate (s) sides are denoted as RA(1)_a,s_ = RA(0,1)_a,s_ = RA(1,0)_a,s_. The second-order RAs are denoted similarly to RA(1,1)_a,s_, with third and fourth orders denoted as RA(2)_a,s_ and RA(2,1)_a,s_ (=RA(1,2)_a,s_), respectively. The wavelengths corresponding to the RAs of the three arrays investigated are given in Table 2. The periodicity of the arrays allows for SLRs to exist; however, for nanoparticle arrays that are fabricated on a glass substrate SLRs would not be able to propagate because of the index mismatch between the substrate and supersubstrate (air).^27,52^

**Table 2:** RA Wavelengths for the Three Arrays

array	RA(1)_a_ (nm)	RA(1,1)_a_ (nm)	RA(1)_s_ (nm)	RA(1,1)_s_ (nm)	RA(2)_s_ (nm)	RA(2,1)_s_ (nm)
A600	600	424.3	900	636.4	450	402.5
A550	550	388.9	825	583.4	412.5	369.0
A480	480	339.4	720	509.1	360	322.0

## 2 QGMs in TiO_2_ Metasurfaces on a TiO_2_ Layer

**Figure 2:**
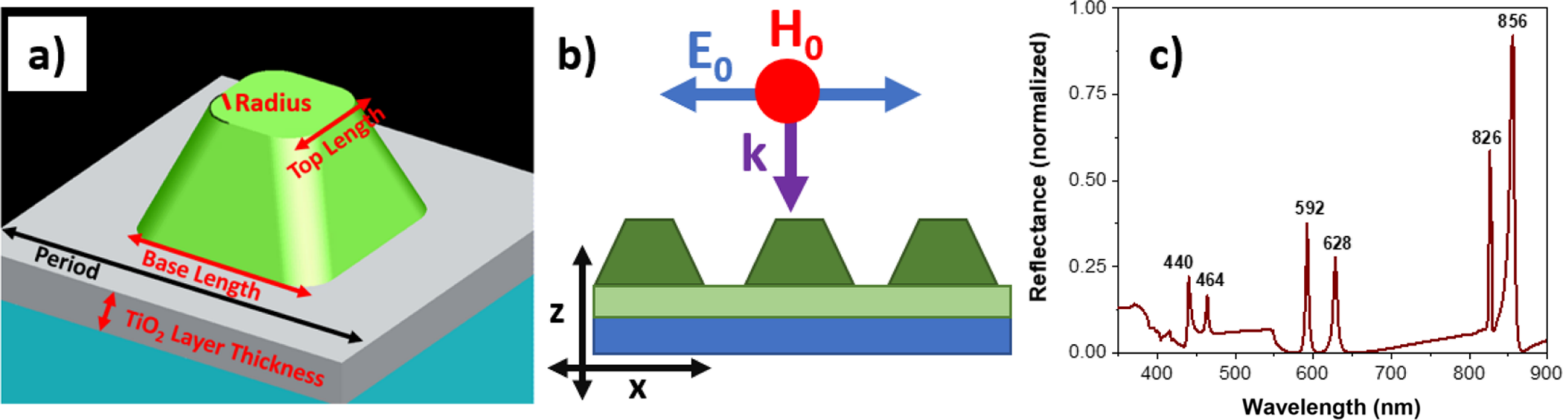
(a) Diagram of geometric array properties used in finite-difference time domain (FDTD) simulations. (b) Schematic of the pillar array with incident plane wave radiation propagating along the *z*-axis with the electric field (with amplitude *E*_0_) linearly polarized along the *x*-axis and the magnetic field (with amplitude *H*_0_) linearly polarized along the *y*-axis. (c) Numerically calculated reflectance spectrum of the A550 array with the peak wavelengths given.

The optical properties of the structures are investigated using FDTD numerical simulations and the conditions under which QGMs can exist will be discussed. Details of the FDTD calculations of the reflectance and transmittance spectra are given in the methods in Section 5 and Figure S1. A schematic of the nanopillar used in the simulations is shown in Figure 2a, with the dimensions taken from Table 1. The pillars of the array were simulated as truncated round pyramids as seen in the SEM images in Figure 1. The radius for the roundness of the pillar is approximately 30 nm. The reflectance spectrum is calculated using a normally incident plane wave polarized along the *x*-axis, Figure 2b. The glass substrate refractive index is *n* = 1.5, the TiO_2_ pillar has a refractive index of 2.1, and the TiO_2_ residual layer has an effective refractive index of 1.9. The refractive index of the pillar was chosen as 2.1 as this is a good approximate static value to the results obtained on the titania coatings for soft-NIL fabrication^46^ and because the layer is more porous and rougher, a slightly lower value was chosen. The impact of porosity on the refractive index is discussed in more detail later (see Figure S5). A calculated reflectance spectrum for array A550 is shown in Figure 2c, with the numbers indicating the peak positions. The reflectance spectrum shows six narrow peaks in pairs, the first pair at 440 and 464 nm, the second pair at 592 and 628 nm, and the last pair at 826 and 856 nm.

**Figure 3:**
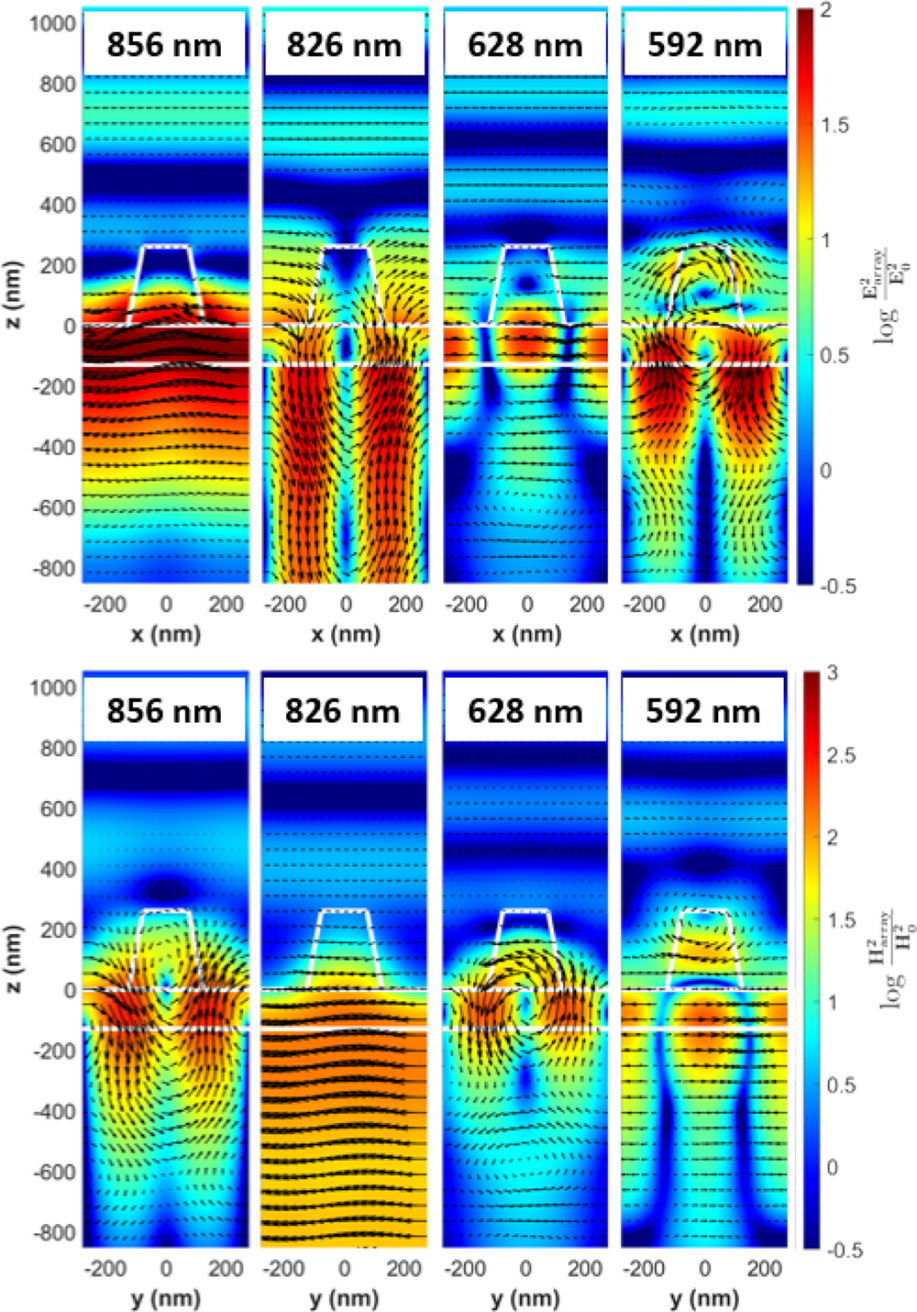
Electric and magnetic field intensity distributions normalized to the incident field intensity for the A550 array. Calculated using a substrate refractive index of 1.5, a TiO_2_ layer of refractive index of 1.9, and a TiO_2_ pillar of refractive index of 2.1. The color represents the intensity on a logarithmic scale, and the black arrows represent the real part of the vector electric/magnetic field in the *xz*/*yz* plane. Top row: electric field maps. Bottom row: magnetic field maps for the wavelengths corresponding to the peaks shown in Figure 2c.

By looking at the magnetic and electric field distribution in the array in Figure 3, it can be immediately seen that for all these peaks, except that at 826 nm, there are high electric and magnetic field intensities in the residual TiO_2_ layer. For the peak at 826 nm, the field penetrates into the substrate, which could be due to its close proximity to RA(1)_s_ (see Table 2 for RA positions for the A550 array). At 856 nm, the electric field intensity distribution shows very intense fields, particularly in the TiO_2_ layer. The magnetic field is also mainly confined in the layer and circulating, a signature of electric modes. By looking at the electric and magnetic field orientation, it can be concluded that there is an electric transverse guided mode in the layer propagating along the y-axis. This mode is coupled into the layer and scattered by the pillars. Therefore, this reflectance peak is due to an electric QGM that is mostly confined in the layer and propagating along the y-axis. This mode is referred to as E-QGM1. At 826 nm, the electric field intensity distribution has a very similar profile to that of the magnetic field at 856 nm. One observes a circulating electric field, and the magnetic field intensity at 826 nm has a very similar shape to the electric field at 856 nm. The orientation of the magnetic field is predominantly along the *y*-axis. This peak is consequently a magnetic transverse mode propagating along the *x* direction in the layer. While this mode is mostly in the substrate, there is a component propagating in the layer, and it is not observed without the presence of the layer. The mode is scattered out by the pillars as a reflectance peak and is referred to as M-QGM1.

The second set of peaks observed in reflectance exhibit field intensity maps that are significantly different from the E-QGM1 and M-QGM1. For the electric and magnetic field intensity distributions for the reflectance peak at 628 nm, it is observed that the fields are confined predominantly in the layer and because the magnetic field forms a loop around the layer, this indicates that the peak at 628 nm is a higher order electric QGM traveling along the y-axis of the layer. Three lobes of intense electric field in the layer can be observed, one directly underneath the pillar and two between the pillars, with electric fields oriented 180° from their neighbors. This is a higher order transverse electric mode traveling along the y-axis in the layer referred to as E-QGM2. Following the same analysis as that for E-QGM2, the peak at 592 nm is a magnetic QGM, referred to as M-QGM2. See Figure S2 for the electric and magnetic fields and the discussion for the third set of peaks at 440 and 464 nm. These are third-order electric and magnetic QGMs and are referred to as E-QGM3 and M-QGM3, respectively. In the following sections, different parameters of the array will be varied to confirm the behavior of the QGMs with respect to the RAs and the thickness of the TiO_2_ layer.

### 2.1 QGM Dependence on the Lattice Constants

**Figure 4:**
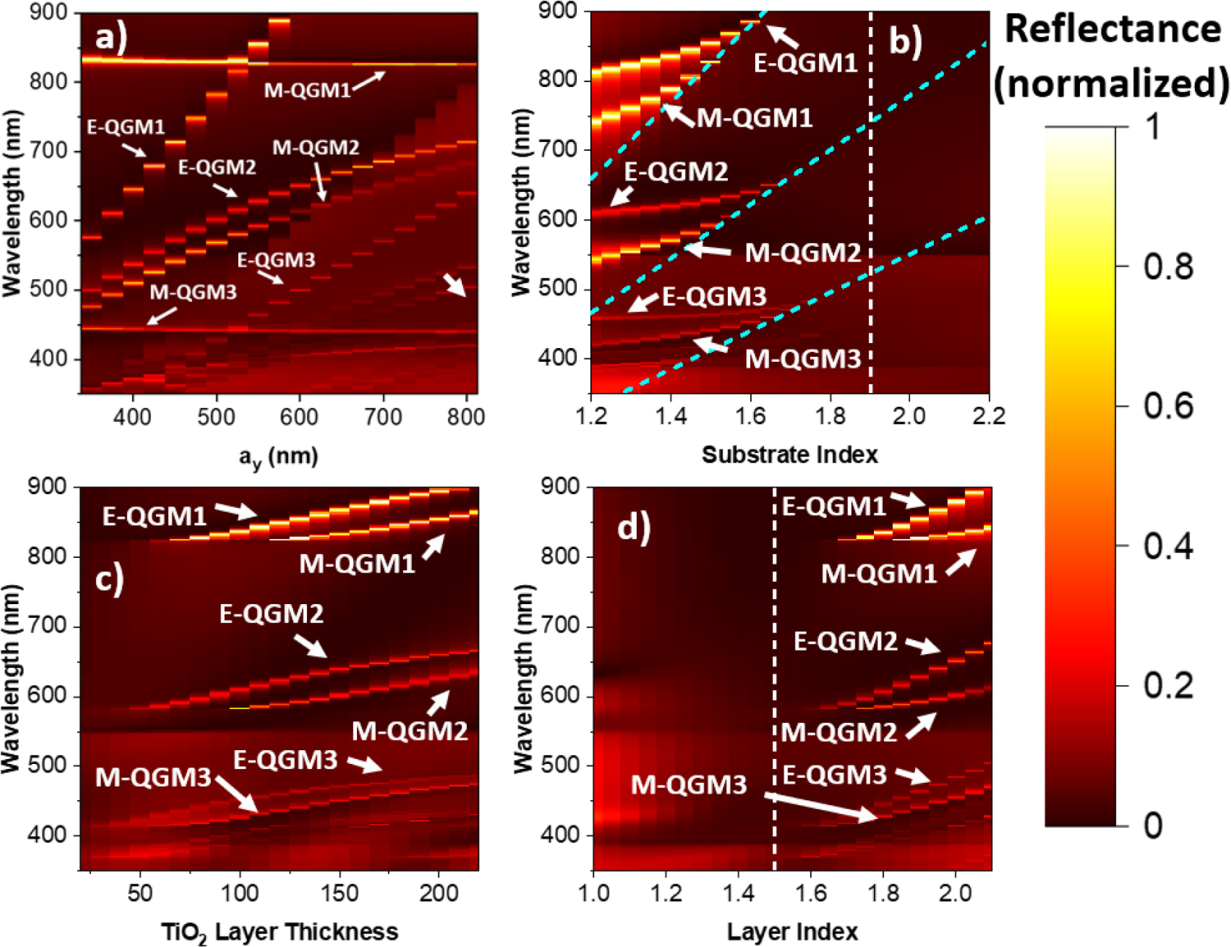
Numerically calculated dependence of the reflectance spectrum on (a) lattice constant *a_y_*, (b) substrate refractive index where the blue dashed lines correspond to the RAs and the vertical white dashed line indicates the refractive index of the residual TiO_2_ layer, (c) TiO_2_ layer thickness, and (d) TiO_2_ layer refractive index with the vertical white dashed line indicating the refractive index of the substrate.

In Figure 4a, the dependence of the reflectance spectrum on the lattice constant *a_y_* is shown. One observes that E-QGM1 varies linearly with the change of the lattice constant *a_y_*, while M-QGM1 remains constant. In contrast, a reciprocal behavior for M-QGM1 and E-QGM1 is observed when the lattice constant in the *x* direction is varied in Figure S3. It is clear now that E-QGM1 is propagating along the *y*-axis and is coupled to the RA(0,1)_s_ from the substrate side (825 nm at *a_y_* = 550 nm) and that M-QGM1 is propagating along the *x*-axis and is coupled to RA(1,0)_s_ from the substrate side (825 nm at *a_y_* = 550 nm). For the second set of peaks, E-QGM2 and M-QGM2, it is observed that both are varying when either *a_x_* or *a_y_* is varied. Furthermore, their dependence on the lattice constants is not linear, but rather has a  dependence, where *c* is a constant. Because of the nonlinear dependence on the lattice constant and their proximity to RA(1,1)_s_ from the substrate side, the E-QGM2 and M-QGM2 are clearly coupled with the RA(1,1)_s_ from the substrate side (583 nm at *a_x_* = *a_y_* = 550 nm). For the last set of peaks, E-QGM3 and M-QGM3, one observes a very similar behavior to the first set of peaks: E-QGM3 is linearly dependent on *a_y_* while being independent of *a_x_*, and M-QGM3 is linearly dependent on *a_x_* while being independent of *a_y_*. The only RA close to these resonances that would explain this behavior is the second-order RA(2,0)_s_ and RA(0,2)_s_ (for *a_x_*, *a_y_* = 550 nm at 412.5 nm). Therefore, E-QGM3 is coupled to RA(0,2)_s_ and propagating along the y direction, and M-QGM3 is coupled to RA(2,0)_s_ and propagating along the *x* direction.

### 2.2 QGM Dependence on the Substrate and TiO_2_ Layer Properties

In Figure 4b, the dependence of the reflectance spectra on the substrate’s refractive index is shown. It can be observed that all QGM positions vary with the refractive index. An asymptotic behavior is observed for all the QGMs as they approach their coupled RA, further confirming their coupling. In Figure 4d, one sees the impact of the refractive index of the layer on the positions of the QGMs. One can also observe that the index of the layer must be larger than that of the substrate to support QGMs. Furthermore, the position of the QGMs can also be tuned by the thickness of the layer (see Figure 4c). The layer needs to be sufficiently thick to support QGMs, and no lower order QGMs are present for very thin layers. The positions of the QGMs are not significantly affected by the pillar’s shape and refractive index (see Figure S4). However, the peaks do become broader as the pillars become more optically thick (higher pillar refractive index), as the QGMs are more rapidly scattered out by the pillar.

## 3 Experimental Reflectance and Transmittance

### 3.1 TiO_2_ Metasurfaces

**Figure 5:**
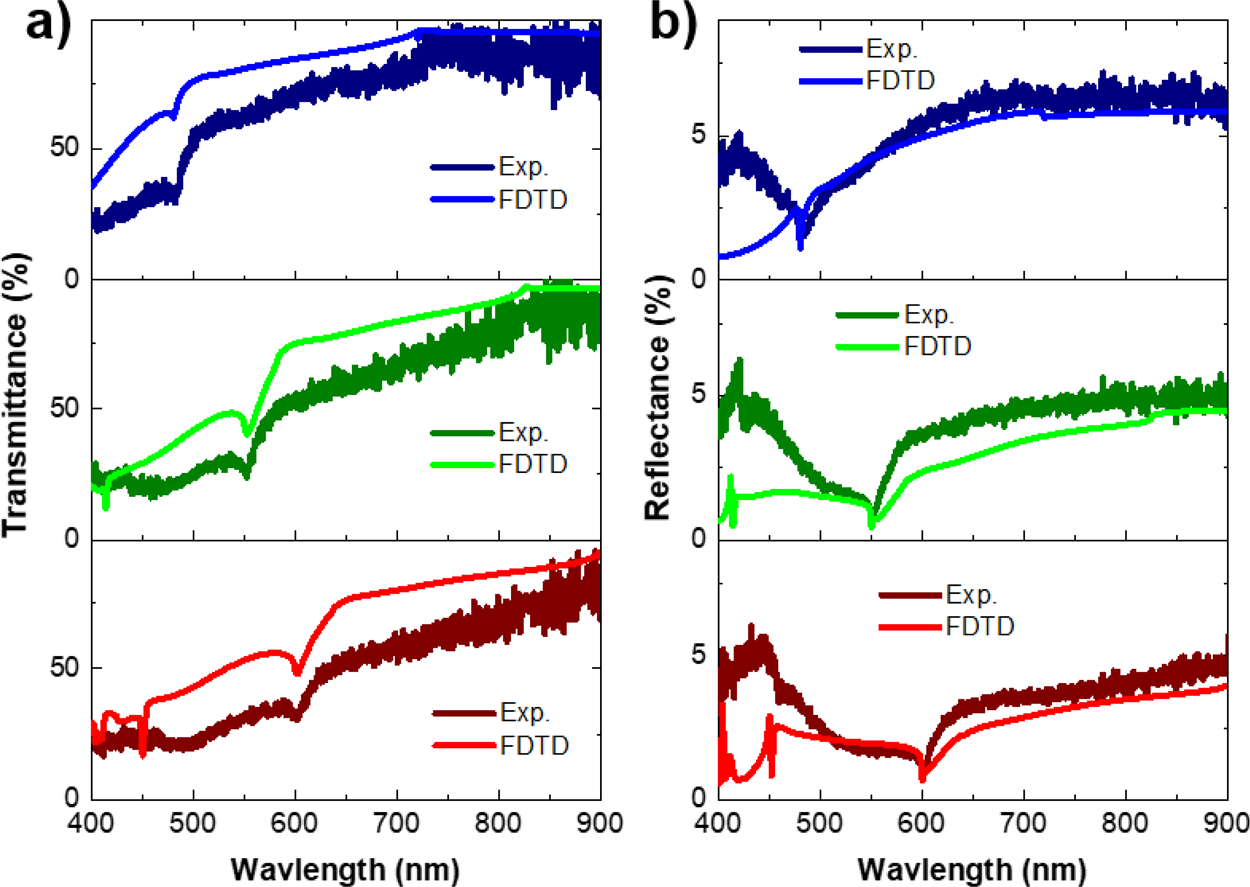
Experimental and FDTD results for (a) normal incidence reflectance and (b) normal incidence transmittance for the A600 (bottom), A550 (middle), and A480 (top) arrays. FDTD simulations consider a 30% porosity for the pillar and 45% porosity for the residual layer with 50 nm root mean square (RMS) surface roughness.

The experimental reflectance and transmittance spectra for the three arrays measured under normal incidence illumination are presented in Figure 5. The measurement setup is described in the experimental methods in Section 5. The reflectance spectra for the three arrays are very similar to each other. It is noted that no sharp peaks are observed, in contrast to the numerically simulated spectrum shown in Figure 2c. This is discussed in detail below. Features at the RA wavelengths can be seen. At RA(1)_a_ (see RA positions in Table 2), one observes a sharp dip in reflectance and at RA(1,1)_s_ one sees a sudden change in slope for all arrays. For the A550 and A480 at RA(1)_s,_ there is a slight change in slope. One does not observe this feature for the A600 because the RA(1)_s_ is at 900 nm, which is the limit of the experimental spectral range. Similar features are observed in the transmittance spectra in Figure 5b, such as at the wavelengths corresponding to RA(1)_a_ and RA(2)_s_, and there is a sudden change in the slope in the transmittance spectrum at RA(1,1)_s_.

The transmittance and reflectance do not show any signs of QGM coupling to RAs. This is due to the refractive index of the TiO_2_ layer being too low because of the high porosity and increased surface roughness of the TiO_2_ layer as seen in Figure 1d–i. To demonstrate the effect of the layer porosity, spectra were simulated using the refractive index of TiO_2_ corresponding to different porosities. Details of the calculation of the refractive index of porous TiO_2_ are given in the methods in Section 5. The calculated refractive indices as a function of porosity are shown in Figure S5. The surface roughness of the TiO_2_ layer is also taken into account and with the RMS of the surface varied to simulate the randomness of the layer. The impact of randomness in the array on the coupling of RAs with particle resonances has been reported by Zakomirnyi et al.^55^ The randomness in the different features of the array elements (period, size, and discontinuities) weakens the collective resonances in the arrays, so one expects to observe similar behavior for RA-coupled QGMs. The spectra for different porosities of the pillar and layer, as well as the effect of roughness of the surface, are shown in Figures S6 and S7, respectively. The parameters which provide the best agreement with the experimental spectra are 30% porosity for the pillar and 45% porosity for the residual TiO_2_ layer with 50 nm roughness, shown in Figure 5. Figures S8 and S9 show that the effect of the cracks in the TiO_2_ layer is well approximated in the simulations by the porosity of the layer.

The shape of the FDTD transmittance spectra in Figure 5a is nearly identical to the experimental transmittance for all the arrays. The FDTD transmittance is slightly higher than the experimental one, which can arise from imperfections on the backside of the glass substrate and unaccounted effects of the imperfections of the TiO_2_ layer and array (e.g., small fluctuations of the sol–gel layer, scattering). The FDTD reflectance spectra above 500 nm in Figure 5b match well in shape and intensity with the experiments for all the arrays. From 400–500 nm, the FDTD results do not reproduce the increase in intensity observed in the experimental data; this feature is missing in the three arrays. This feature was not reproduced by any combination of refractive index for the pillar and layer. It is possible that it is an artifact from higher order transmission that reflects from the lower substrate air interface and is detected within the collection angle in the experimental setup.

### 3.2 Optical Properties of the Arrays with Incorporated R6G

The porosity of the residual TiO_2_ layer provides the possibility to incorporate another material. This can increase the refractive index of the layer and result in the appearance of distinctive QGM resonances in the reflectance and transmittance spectra.

R6G is chosen as it enables modification of the real refractive index and introduces absorption in a different spectral range allowing us to probe the effect of both on the QGMs. The absorption peak is in the range of 500–530 nm overlapping with the RA(1,1)s of array A480 at 509.1 nm, while for arrays A600 and A550 RA(1,1)s, at 636.4 and 583.4 nm, respectively, is far from the absorption peak. This allows for control of the coupling of different-order QGMs, as QGMs will not be allowed to propagate if losses are high because of the R6G absorption.

**Figure 6:**
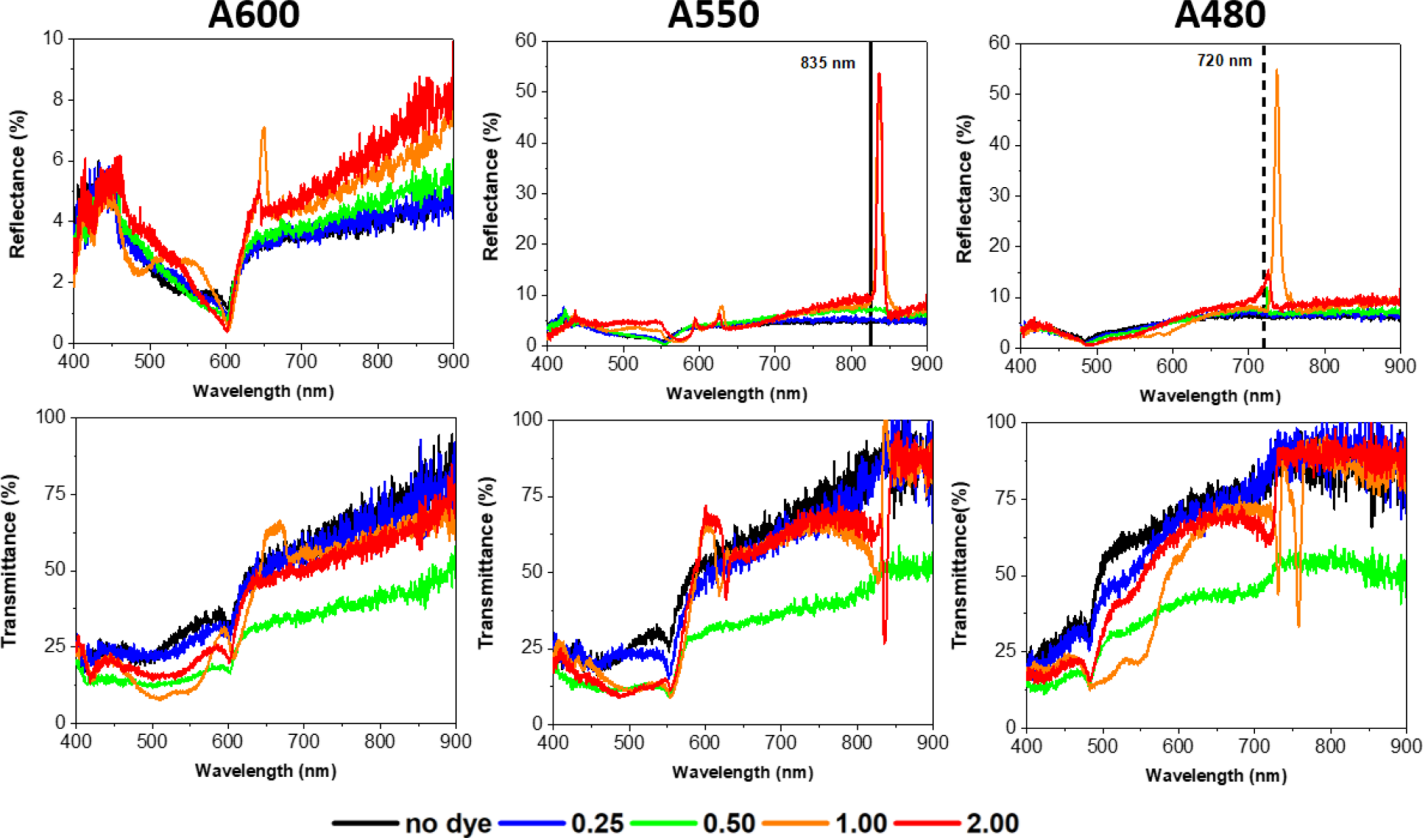
Dependence of the experimental reflectance (top row) and transmittance (bottom row) on increasing R6G concentration on the A600, A550, and A480 arrays. The black dashed line shows the position of RA(1)_s_. The concentrations are given in mM.

Four concentrations of R6G in ethanol were spin-coated onto the samples: 0.25, 0.50, 1.00, and 2.00 mM, as described in the methods in Section 5. The effect of the R6G on the optical properties of the array was reversible; after cleaning the sample, the reflectance and transmittance spectra returned to those of the bare array. The influence of R6G on the reflectance and transmittance spectra of the arrays can be seen in Figure 6. Very sharp and strong reflectance peaks at high R6G concentrations emerge in the spectra for the A550 and A480 arrays. The features of the experimental spectra will be explained first, followed by numerical simulations to confirm the presence of the QGMs after the incorporation of R6G dye into the arrays.

The effect of R6G on the A550 array will be discussed first, as it shows the most features. The two lower R6G concentrations (0.25 and 0.50 mM) yielded virtually no changes in the reflectance spectra (Figure 6). For the higher concentrations, one can observe a very strong reflectance peak at 835 nm very near RA(1)_s_ at 825 nm. This peak likely results from an electric QGM coupled RA(1)_s_ (E-QGM1). The sharp peaks at 594 and 626 nm are not nearly as strong in reflectance as the E-QGM1 peak, that is, less than 10%. In the simulation, they are usually approximately half of the intensity of the E-QGM1, as seen in Figure 2. From the Fano-shape of these peaks and their close position to the RA(1,1)_s_, these are attributed to the electric and magnetic QGM coupled RA(1,1)_s_ that is, E-QGM2, M-QGM2, with E-QGM2 at 626 nm, and M-QGM2 at 594 nm. The RA(1)_a_ feature at 550 nm is present for all concentrations and does not change in position. This correlates to the R6G being present mostly in the layer and possibly forming a thin layer on top. If the R6G layer was too thick, the RA(1)_a_ would be lost and a new RA(1) related to the refractive index for the thick R6G layer would appear. One can also observe a small feature related to RA(2)_s_ become slightly more prominent and spectrally redshifted. The redshift is caused mainly by an increasing refractive index in the layer but can also occur by increasing the layer thickness. Significant changes in transmittance are also observed at the higher R6G concentrations. For 2.00 mM, there are dips in transmission at 628 and 835 nm corresponding to the E-QGM2 and E-QGM1 peaks observed in reflectance, respectively. The reflectance and transmittance in the region between 450 and 550 nm are lower because of the absorption of the R6G, see Figure S10a. For 1.00 mM, the transmittance spectrum does not correspond well with the reflectance spectrum owing to the uneven spread of R6G (see images in Figure S11 at 1.00 mM). Despite this, the spectrum is interesting as it shows that the region measured has a slightly lower concentration of R6G, resulting in a layer with a lower refractive index; the E-QGM2 dip blueshifts and, similarly, the dip at E-QGM1 is not nearly as strong and is blue shifted to the RA.

Similar observations are made for the A480 array. Again, no QGMs were observed for the lower concentrations. However, for the 1.00 mM concentration one observes a sharp peak in reflectance at 736 nm, attributed to an electric QGM coupled to RA(1)_s_ (E-QGM1). No QGM coupling to the RA(1,1)_s_ is observed because the RA(1,1)_s_ is at 509 nm which corresponds to a region of high absorption by R6G which prevents the formation of a QGM. In the transmittance spectra, one notices two dips at 730 and 757 nm, attributed to the M-QGM1 and E-QGM1, respectively, as the magnetic QGM occurs at shorter wavelengths than the electric QGM and because of their proximity to RA(1)_s_ and sharp Fano lineshape. One can also estimate the increased concentration of R6G on the A480 array at 1.00 mM from the dip in transmission between 500 and 550 nm, which is a direct consequence of high R6G concentrations.

For the A600 array, the experimental spectral range does not extend to wavelengths higher than 900 nm; therefore, QGMs coupled to RA(1)_s_ cannot be observed. For 1.00 mM, there is a peak at 647 nm, which is quite close to the RA at 636 nm and attributed to the E-QGM2. The refractive index of the layer is not high enough to support M-QGM2. For the same reasons as in the previous arrays, one does not observe any QGMs coupled to RA(2)_s_. None of the other concentrations show any QGMs. The transmittance spectra for the A600 at 1.00 mM do not quite match with the reflectance following the trend of the previous arrays at this concentration because of the uneven dye distribution. Based on the feature observed around 650 nm, the A600 array might be supporting E-QGM2 and M-QGM2; however it is not conclusive.

### 3.3 Confirmation of QGMs by FDTD

**Figure 7:**
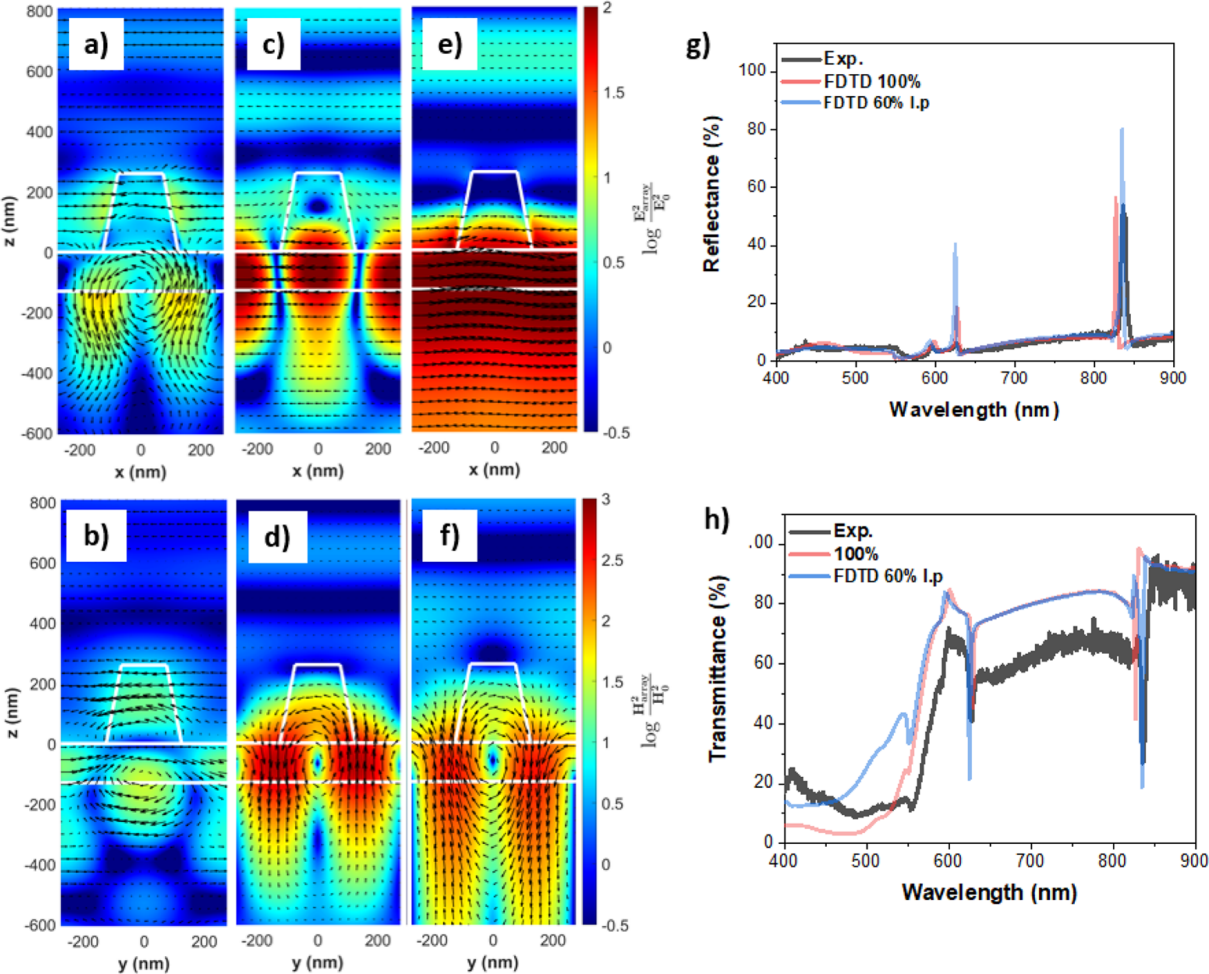
Electric and magnetic field intensity distributions normalized to the incident field intensity for the A550 array with R6G incorporated into the residual TiO_2_ layer (denoted as 60% l.p.). The color represents the intensity on a logarithmic scale and the black arrows represent the real part of the vectorial electric/magnetic field on the *xz*/*yz* plane. (a) Shows the electric field intensity distribution and (b) magnetic field intensity distribution for M-QGM2 at 594 nm. (c) Shows the electric field intensity distribution and (d) magnetic field distribution for E-QGM2 at 628 nm. (e) Shows the electric field intensity distribution and (f) magnetic field intensity distribution for E-QGM1 at 835 nm. Experimental spectra for the arrays with a R6G concentration of 2.00 mM and FDTD simulation spectra for different TiO_2_/R6G ratios: (g) reflectance and (h) transmittance.

In Section 2, it was shown that the QGMs are most strongly influenced by the properties of the layer and the substrate. To investigate the effect of the R6G, it is assumed in the simulation that the R6G is only affecting the TiO_2_ layer, that it penetrates the layer uniformly, and that the layer is flat. The effective refractive index for a mixed layer of R6G and TiO_2_ was calculated using the Maxwell–Garnett equation as described in the methods in Section 5. The spectra calculated for 100% (55% TiO_2_ porosity and 100% R6G concentration) and 60% l.p. (45% TiO_2_ porosity and 60% R6G concentration) provide good agreement of the spectral shape with the measured reflectance and transmittance spectra of array A550 for a R6G concentration of 2.00 mM, see Figure 7g,h, respectively. The peak positions in FDTD reflectance spectrum are a slightly better match for the 60% l.p. refractive index. It is noted that the peak at 626 nm is larger than in the experimental spectrum. This could be due to a higher concentration of R6G being present or due to the tail of the R6G absorption on the arrays being slightly longer because of formation of j-aggregates on the arrays. The peak of the j-aggregate absorption is roughly at 550 nm.^56^ The features of the sharp dips in the transmittance spectra shown in Figure 6h also agree well with the experimental results, and the overall spectral shape matches very well with the experimental results, except in the region between 450 and 550 nm. The shape of this region in the experimental spectrum is mainly caused by the absorption of R6G in this region. The 100% R6G concentration layer matches with this spectral region better as it has a higher R6G concentration than the 60% l.p. layer. These relative ratios of TiO_2_ to R6G give good correspondence with the experimental data and can reproduce all the main spectral features; however, it is also possible to obtain similar agreement using a higher concentration of R6G and different porosity ratios.

To confirm that the sharp peaks observed in reflectance in the FDTD simulations are QGMs, the field maps for the array with a layer 60% l.p. can be compared with those for the TiO_2_ arrays with no porosity shown in Figure 3. The electric and magnetic field intensity distributions for the M-QGM2 at 594 nm can be seen in Figure 7a,b, respectively. Similarly, for the E-QGM2 at 624 nm shown in Figure 7c,d, the field maps correspond to an electric QGM propagating along the x-axis, which is coupled to RA(1,1)_s_. The electric and magnetic field profiles for the E-QGM1 at 835 nm are shown in Figure 7e,f, respectively. The simulations confirm that the sharp reflectance peaks and transmission dips observed with R6G are directly related to QGMs coupled to RAs. However, no higher order QGMs are observed for RA(2)_s_ because of high losses from the R6G absorption in the layer in the range from 400 to 580 nm.

There have only been a few observations QGMs in titania-based structures.^57,58^ This is the first report in which the electric and magnetic QGMs coupled to the mixed RA(1,1) have been observed. Furthermore, the E-QGM1 mode observed for the A550 and A480 at high R6G concentrations had Q-factors of ∼110 (fwhm ∼8 nm) and ∼ 80 (fwhm ∼8 nm), respectively, and a reflectance peak of roughly ∼50%. The experimental Q-factors for the A550 arrays are considerably lower than the values from the FDTD calculation using the “60% l.p.” index for the layer, resulting in a Q-factor of ∼ 280 and reflectance peak of ∼80%. The difference between experiment and simulation is very likely because of unaccounted factors in the simulation, such as an increase in the refractive index of the pillar’s because of the addition of R6G, irregularities in the residual layer’s thickness, or imperfections of the array. The calculated FDTD values are comparable to the Q-factors obtained from measurements of Murai et al. on SLRs in silicon rectangular arrays; they obtained a Q-factor of ∼298 (fwhm ∼2 nm) and a high extinction of 90% for arrays with a smaller particle size.^24^ The sharpness of the QGM peaks in the arrays measured in this work can be improved in these arrays by having pillars of smaller dimensions and by incorporating different materials with less losses into the layer to increase the index. Despite the collective resonances measured in the arrays presented in this work not being as high as previously reported values, the porosity of the residual layer allows for materials to be incorporated into the layer. This allows for reversible manipulation of the QGMs. Nanoparticle arrays supporting SLRs are typically easier to use for light–matter interaction as the regions of enhanced field are more accessible for the placement of dyes, quantum dots, or other emitters. The use of QGMs is more challenging because of the difficulty in accessing regions of enhanced field intensity within the waveguide. The fact that the QGMs manifest arising from the infiltration of the R6G into the layer shows that the incorporated material will be able to access the regions of high electric field intensities within the layer (see Figures 7c,e and 3). This opens the way for further exploration of the application of QGMs for enhanced light–matter interaction such as enhanced emission and lasing. Furthermore, the materials incorporated into this layer, as demonstrated, can change the optical properties of the layer which affects the formation and positions of the QGMs making these arrays good candidates for applications in sensing.

## 4 Conclusions

QGMs in TiO_2_ metasurfaces have been investigated, and reversible control of the QGMs has been experimentally demonstrated. The TiO_2_ metasurfaces fabricated via soft-NIL can support QGMs if the refractive index and thickness of the underlying TiO_2_ layer are sufficiently high. Simulations showed that the fabrication dimensions would support QGMs, but because of the porosity of the layer no QGMs were observed for the fabricated arrays in the normal reflectance and transmittance measurements. However, the porosity allows the properties of the layer to be easily tuned by incorporating different materials to modify the effective refractive index. It was shown that by the incorporating R6G onto the arrays the QGMs can be observed. The presence of QGMs was verified by inspection of the calculated electric and magnetic field maps. Furthermore, this process is reversible. The TiO_2_ layer is a consequence of the soft-NIL fabrication process, removing the need for additional fabrication steps to incorporate a waveguide into the structure. It can be used to manipulate the optical modes of the TiO_2_ metasurface and can potentially be exploited in a range of applications benefiting from enhanced light–matter interaction.

## 5 Experimental Section/Methods

### 5.1 FDTD Calculation of Normal Reflectance and Transmission Measurements

To calculate the experimental reflectance and transmittance spectra from FDTD simulation, one must consider the back reflectance from the glass substrate (*n* = 1.5), namely, of 4% and the collection angle of the experimental setup. Two simulations are needed, because simulations do not account for the back reflectance of the substrate because the substrate is considered as infinitely thick: one simulation where the incoming beam comes from the air side of the array and another from the substrate side, as shown in Figure S1. Furthermore, only zero-order reflectance and transmittance were analyzed in the simulations to account for the small collection angles in the experiment. From the simulation from the air side, one can obtain the zero-order reflectance *R* and transmittance *T*. From the simulation from the substrate side, one obtains the zero-order reflectance R* and transmittance T*. From these, the total reflectance *R_T_* and the total transmittance *T_T_* are calculated using:
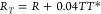
2
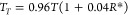
3

The simulations are performed with a 2 nm resolution.

### 5.2 Normal Reflectance and Transmittance Measurements

A custom-built experimental setup was used for the normal incidence and collection reflectance and transmittance measurements. This setup allows for collection of light reflected and transmitted from a sample area of ∼200 μm^2^. A xenon lamp was used as an illumination source, and a very good collimated white light beam was formed. A 50:50 beam splitter was used to direct the collimated white light beam toward the sample and to allow the collection of the reflected light. The reflection and transmission segments are mirror images of each other, and both use the optics in the same manner. Light from the sample is collected by a 15 cm focal length 2.54 cm diameter lens placed 15 cm away from the sample. An iris with a 0.5 cm opening is placed 12.5 cm away from the sample to reduce the collection angle; this yields a collection angle of 1.2°. A series of lenses are then used to form a magnified image onto an iris where a collection area can be selected by moving the sample stage. The light then is coupled into a fiber via a fiber coupler directing the transmitted light into an Andor 230i spectrometer with an Andor CCD camera. The resolution is 1 nm.

To obtain the percent of the total light reflected, first all spectra collected have been corrected by the background light collected when the white light beam was blocked. The reflected light from the arrays was normalized to the reflectance of a clean microscope glass slide and then multiplied by the reflectance of glass at normal incidence which is approximately 8% accounting for both sides of the glass slide. To obtain the percent of transmitted light through the array, the transmitted light was normalized to the transmission when no sample was present.

### 5.3 Refractive Index of TiO_2_ and R6G

Experimentally obtained data for the refractive index on a thin TiO_2_ layer from Sarkar et al.^36^ were used as a reference to calculate different TiO_2_ porosities. The refractive index of TiO_2_ for different porosities^54^ is calculated using the Maxwell–Garnett equation:
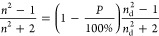
4where *n* is the refractive index, *n*_d_ is the refractive index of bulk TiO_2_ (roughly 2.54 at 550 nm), and *P* is the porosity. The values obtained by Sarkar et al.^36^ are found to correspond to a porosity of ∼15%. The calculated refractive indices for other porosities are shown in Figure S5.

Experimental absorption of R6G on a section of flat TiO_2_ layer next to the arrays (Figure S10a) was used to calculate the n and k values (Figure S10b**)** using a MATLAB code from Djorovic et al. that takes the experimental absorption cross section and calculates the polarizability by performing a Kramers–Kronig transformation.^59^

The effective refractive index for a mixed layer of R6G and TiO_2_ was calculated using the Maxwell–Garnett equation.
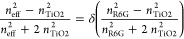
5where *n*_eff_ is the effective refractive index, *n*_R6G_ is the refractive index of R6G, *n*_TiO2_ is the refractive index of TiO_2,_ and δ is the is the porosity of TiO_2_. The *n* and *k* spectra for a range of volume fractions are shown in Figure S12. Because the concentration of R6G and porosity of TiO_2_ in the residual layer and R6G concentration are unknown, different combinations of TiO_2_ porosity and R6G are tested. The different refractive indices for R6G were calculated using eq 4 concentrations from 0 to 100%, where the R6G concentration is 100 % – *P*.

### 5.4 Deposition of R6G

Solutions of four concentrations of R6G in ethanol were prepared: 0.25, 0.50, 1.00, and 2.00 mM. R6G was added to the arrays by dropping 125 μL of the solution onto the arrays and let to rest for 30 s before spin coating. Before spinning on a higher concentration, the arrays were cleaned in acetic acid for 10 min, then for 10 min with acetone, then rinsed in IPA, and dried.

## Supporting Information Available

Diagram showing the calculation of transmittance and reflectance, electric and magnetic field intensity distributions, dependence of the reflectance spectrum on the lattice constant and pillar properties, refractive index of TiO_2_, dependence of the reflectance on porosity, roughness, and cracks, refractive index of R6G, darkfield spectra of the R6G coated metasurfaces as a function of concentration, and effective refractive index of the porous TiO_2_/R6G layer (PDF)
