# Supplementary material for: Quasi-Guided Modes in Titanium Dioxide Arrays Fabricated via Soft Nanoimprint Lithography
Source: ACS Appl Mater Interfaces. 2021 Sep 30;13(40):47860–70. doi: 10.1021/acsami.1c11456 (PMC8517955; doi:10.1021/acsami.1c11456)
Supplement: Supplementary file 1 — am1c11456_si_001.pdf [file am1c11456_si_001.pdf]

Supporting Information

Quasi-Guided Modes in Titanium Dioxide Arrays Fabricated via Soft Nano-Imprint Lithography

*Jorge A. Garcia[1], Calin Hrelescu[1], Xia Zhang[1], David Grosso[2], Marco Abbarchi[2] and A. Louise Bradley[1] **

[1]School of Physics and CRANN, Trinity College Dublin, Dublin 2, Ireland
[2]CNRS, Aix-Marseille Université, Centrale Marseille, IM2NP, UMR 7334, Marseille, France

E-mail: bradlel@tcd.ie

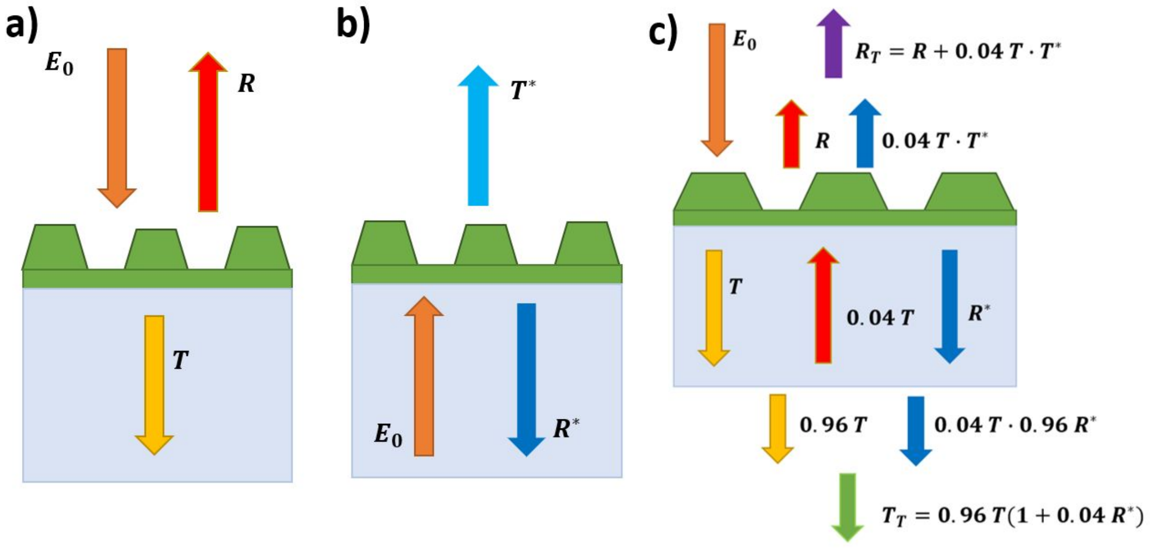

Figure S1. Diagram showing the reflectance R and transmittance T obtained from FDTD simulation with incident field E_0 from the air side, b) diagram showing the reflectance R^* and transmittance T^* obtained from FDTD simulation with incident field E_0 from the substrate side. c) diagram showing the different contributions to the total reflectance R_T and total transmittance $T_{T.}$

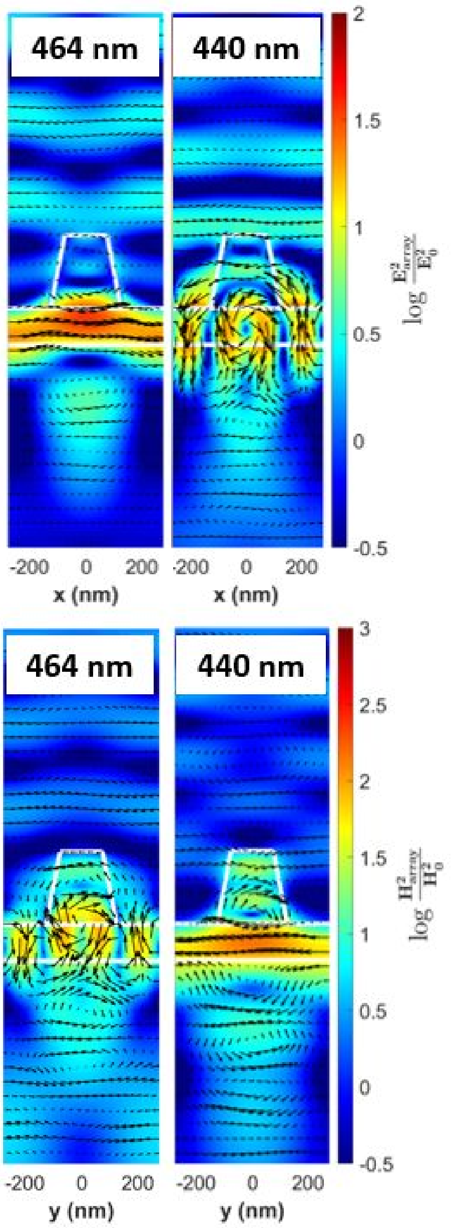

Figure S2. Electric and Magnetic field intensity distributions normalized to the incident field intensity for the A550 array. Calculated using a substrate refractive index of 1.5, TiO_2 layer of refractive index of 1.9 and TiO_2 pillar refractive index of 2.1. The colour represents the intensity on a logarithmic scale and the black arrows represent the real part of the vector electric/magnetic field in the xz/yz plane. Top row: electric field maps, Bottom row: magnetic field maps for the wavelengths corresponding to the third set of peaks shown in **Figure 2(c)**.

Discussion of Figure S2

At 464 nm the electric field is mainly oriented along the x axis and is confined in the layer. The magnetic field is looping around the layer clearly indicating an electric QGM propagating

along the x axis. As seen in **Figure 3(a)** and **Figure S3** it is dependent on a_y and independent of a_x. This mode will be referred to as E-QGM3. Finally, inspection of the electric and magnetic field intensity maps at 440 nm suggests a magnetic QGM propagating along the x axis. This mode will be referred to as M-QGM3.

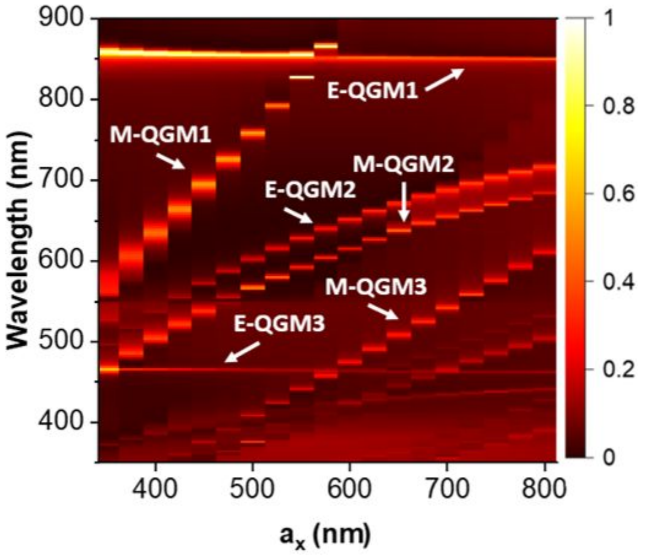

Figure S3. Numerically calculated dependence of the reflectance spectrum on the lattice constant a_y

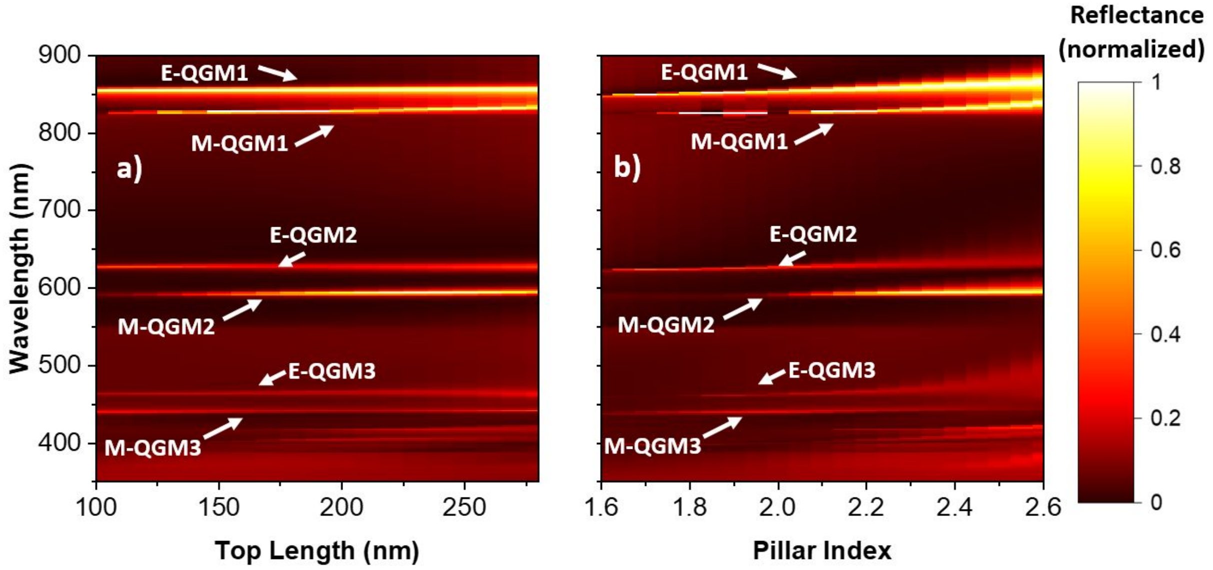

Figure S4. Numerically calculated dependence of the reflectance spectrum on a) the top length of the pillar and b) the refractive index of the pillar.

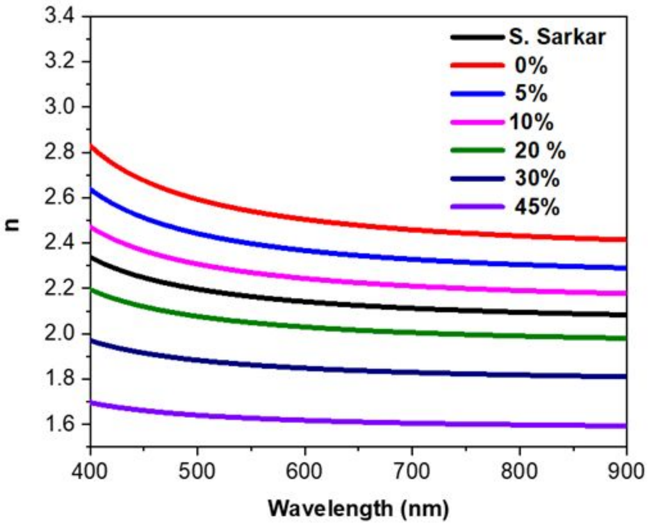

Figure S5. Refractive index of TiO_2 for different porosities used in the FDTD simulations. The line labelled S. Sarkar refers to the data from reference 47 in the paper which was experimentally measured on a layer of TiO_2.

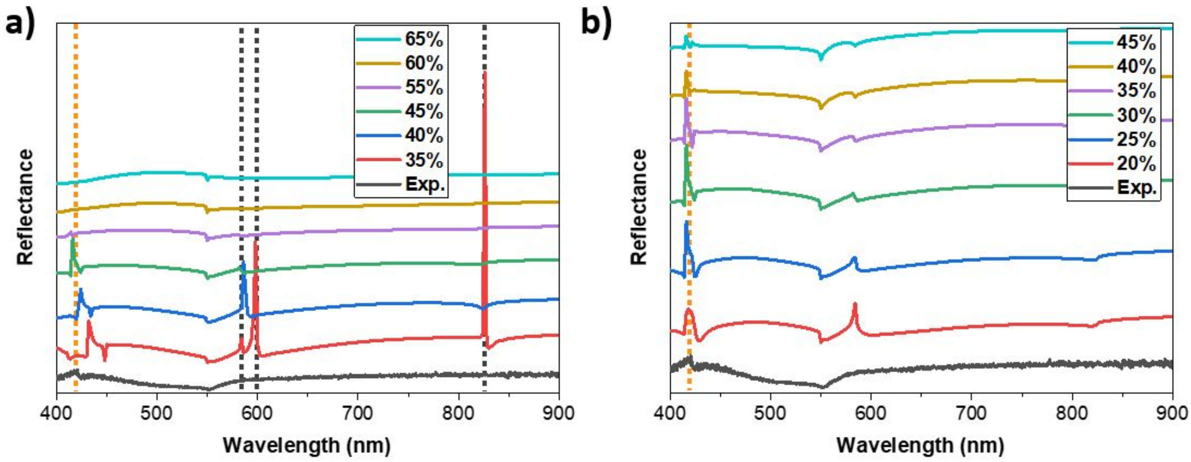

Figure S6. Reflectance spectra obtained from FDTD simulations of the A550 array with a flat layer. a) Dependence on the porosity of the layer with a 30% porosity pillar. The black dashed lines show the positions of the sharp peaks at 35% porosity. The orange dashed line is at $RA(2)_s$. b) Dependence on the porosity of the pillar with a 45% porosity layer. For a 45% layer porosity the feature at $RA(2)_s$ matches best with experimental data. 30% porosity for the pillar gives a good approximation for the experimental reflectance shape.

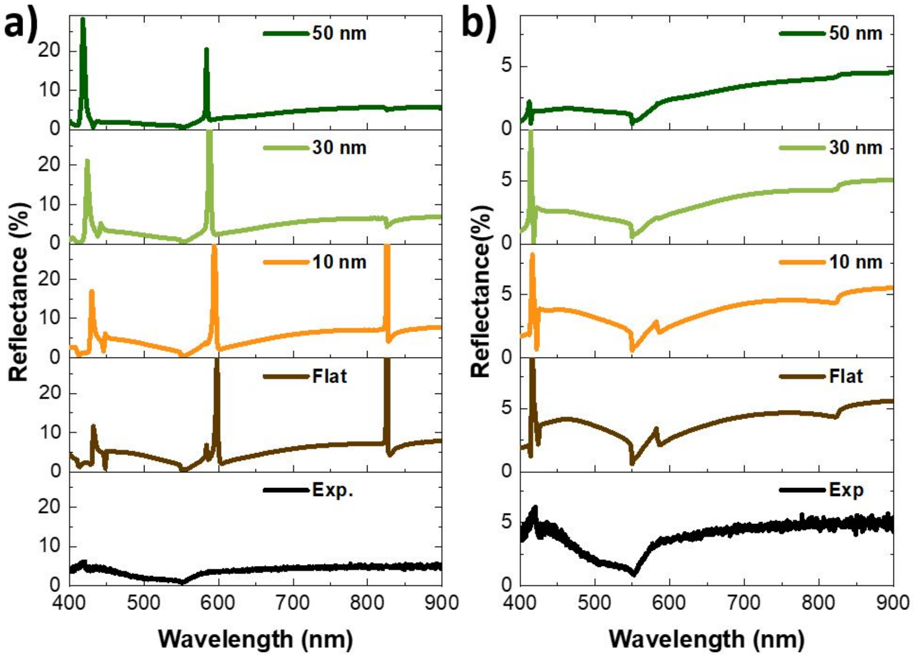

Figure S7. a) Effect of increasing surface roughness (given as a root mean square value) on the reflectance spectra for the A550 array with 30% porosity pillar and 35% porosity layer. b) Effect of increasing surface roughness on the reflectance spectra for the A550 array with 30% porosity pillar and 45% porosity layer. As the roughness increases the effective refractive index of the layer decreases causing a blue shifting QGM peaks and the sharp dips related to RAs from the substrate side become less prominent.

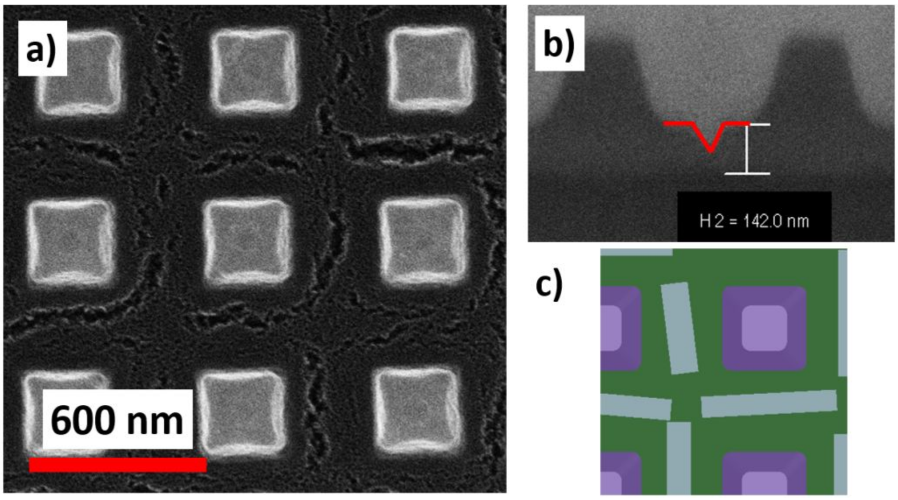

Figure S8. a) SEM image of the A600 array where the gaps between the arrays can be appreciated. b) SEM image of the cross-section of the A600 array where the gap between the pillars is outlined by the red lines and is roughly 80 nm deep. c) Schematics of 3x3 unit cell used for FDTD simulation, the purple squares are the pillars, and the white rectangles are the gaps.

The cracks on the TiO2 layer are between 50 to 100 nm wide and roughly 70-90 nm deep, as can be seen in **Figure S8 a and b**. Simulations were done to assess the effect of the cracks/pores by approximating them by 80 nm deep trenches and with widths of 70 to 100 nm. These cracks were randomly placed over a 3x3 array, as shown in **Figure S8c**. The simulated spectra shown in **Figure S9** show that the effect of the cracks is to lower the effective refractive index of the TiO_2 layer. The QGMs can continue to be observed until the porosity becomes sufficiently high that the effective index of the layer is too low to support them.

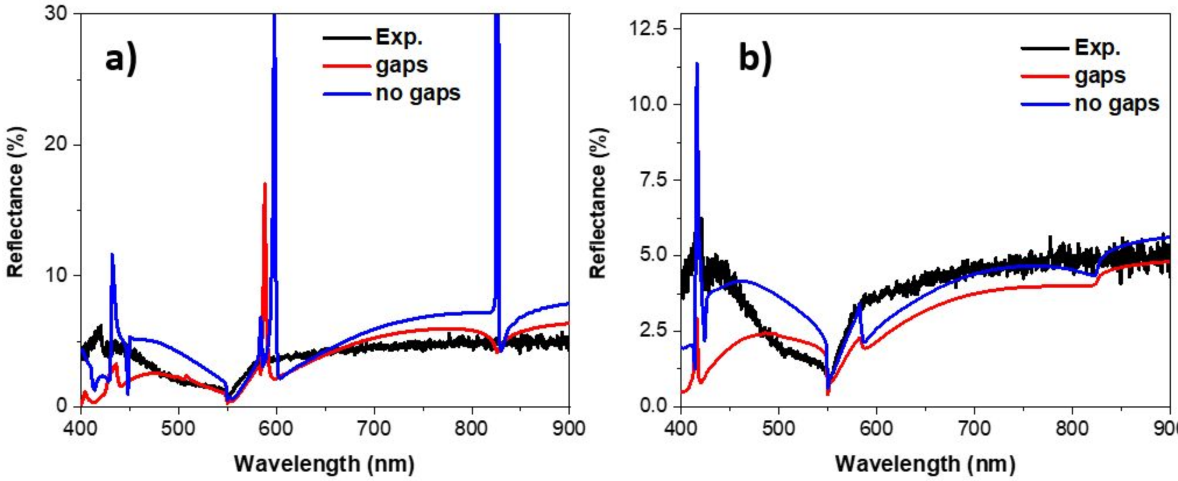

Figure S9. Influence of gaps in the TiO_2 layer on the reflectance spectra of the A550 array with 30% porosity pillar and a layer of a) 35% porosity and b) 45% porosity.

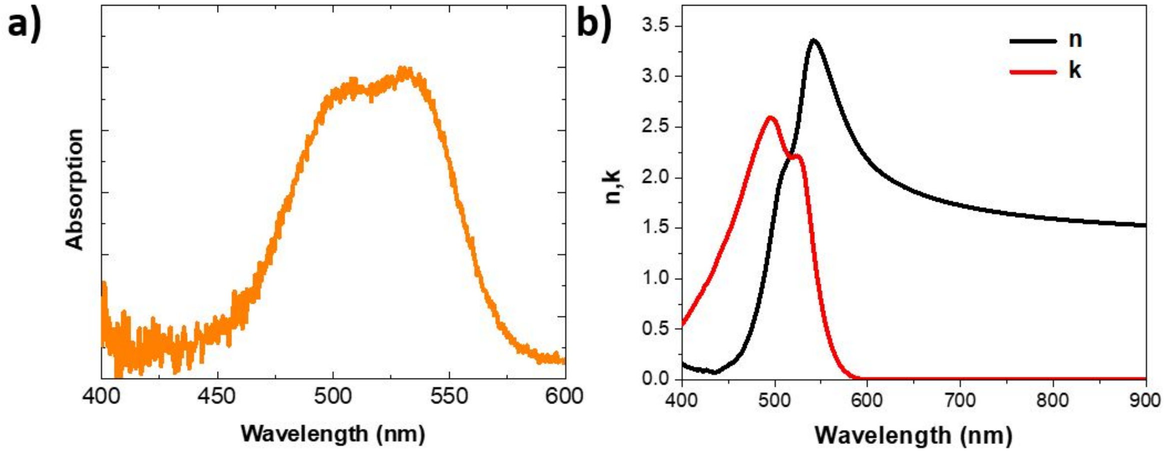

Figure S10. a) Experimental absorption of R6G on a section of flat TiO_2 layer next to the arrays. The monomer and h-dimer absorption peaks can be observed at 530 and 500 nm, respectively. b) Real (n) and imaginary (k) refractive index calculated from the experimental data in a) using a MATLAB code from Djorovic et al[1] that takes the experimental absorption cross section and calculates the polarizability by performing a Kramers-Kronig transformation.

(1) Djorovic, A.; Meyer, M.; Darby, B. L.; Le Ru, E. C. Accurate Modeling of the Polarizability of Dyes for Electromagnetic Calculations. *ACS omega* **2017**, *2* (5), 1804–1811.

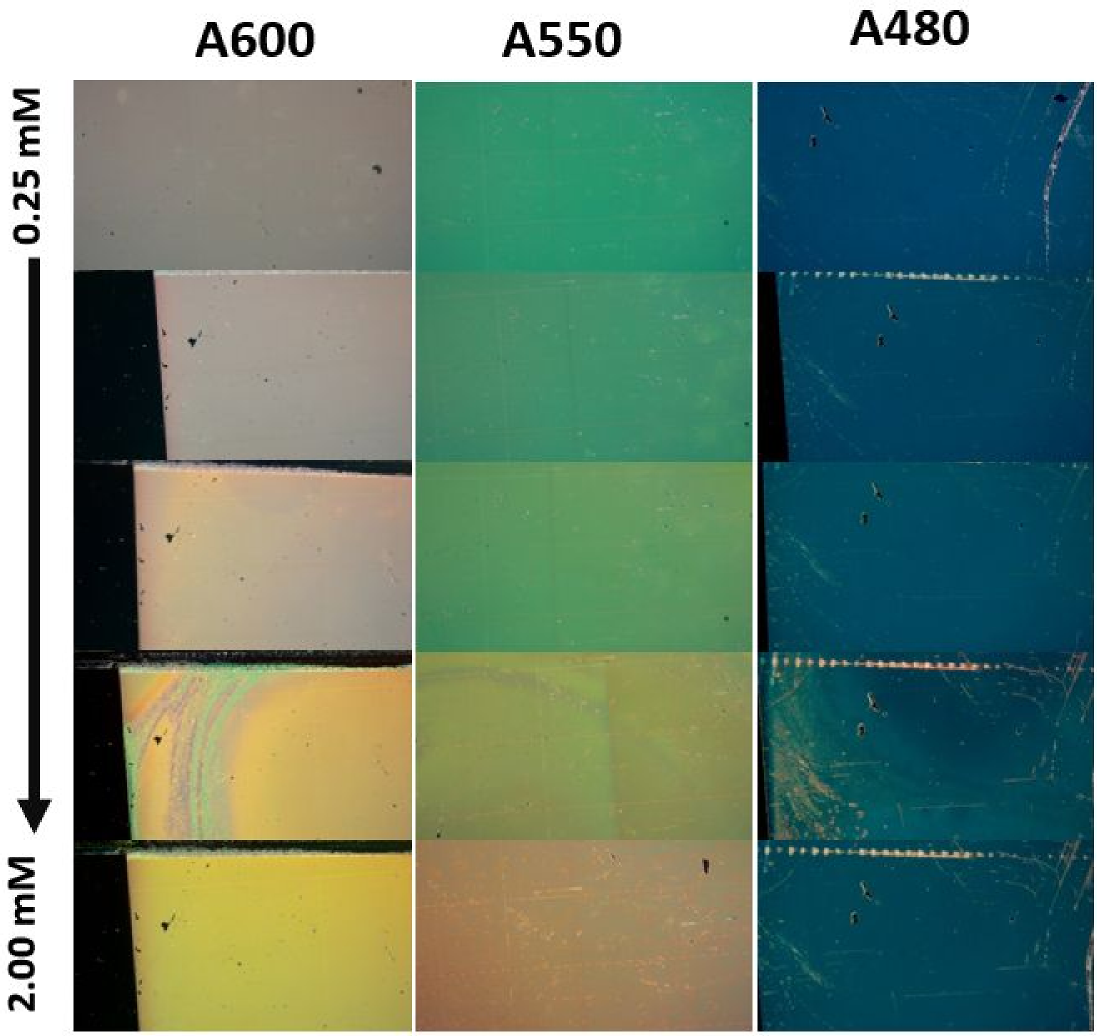

Figure S11. Dark field scattering spectra recorded using a 20x objective for the A600, A550 and A480 arrays as a function of increasing R6G concentration.

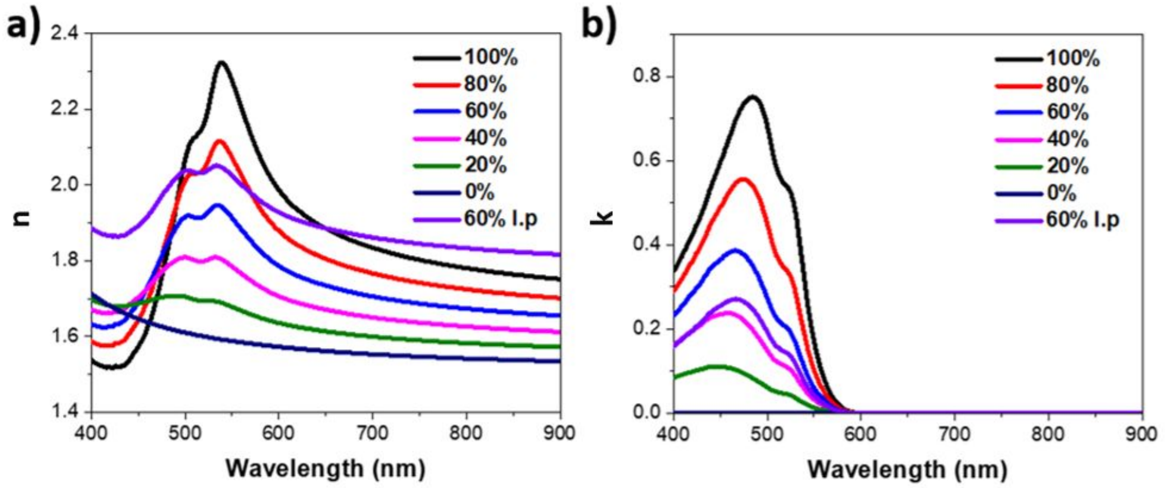

Figure S12. a) Real part and b) imaginary part of the effective refractive index from the TiO_2/R6G mixed layer with increasing R6G concentration (0-100%) of the R6G refractive index in **Figure S8** for TiO_2 porosity $\delta = 0.55$. The label "60% l.p" refers to 60% concentration R6G for a TiO_2 porosity $\delta = 0.45$.
